# Effect of Two-Stage Water Addition on Consistency of Processed Cheese: Physicochemical, Mechanical, Thermal, and Organoleptic Approach

**DOI:** 10.3390/foods14081361

**Published:** 2025-04-15

**Authors:** Anna Vincová, Martina Polášková, Martin Stěnička, Kristýna Šantová, Vendula Kůrová, Barbora Lapčíková, Tomáš Gryger, Eva Lorencová, Zuzana Lazárková, Agnieszka Pluta-Kubica, Markéta Pětová, Ondřej Novosad, Richardos Nikolaos Salek

**Affiliations:** 1 Department of Food Technology, Faculty of Technology, Tomas Bata University in Zlin, Nám. T. G. Masaryka 5555, 760 01 Zlin, Czech Republic; a_vincova@utb.cz (A.V.); k_santova@utb.cz (K.Š.); v_kurova@utb.cz (V.K.); lapcikova@utb.cz (B.L.); t_gryger@utb.cz (T.G.); lorencova@utb.cz (E.L.); lazarkova@utb.cz (Z.L.); 2 Department of Polymer Engineering, Faculty of Technology, Tomas Bata University in Zlin, T. G. Masaryka 5555, 760 01 Zlin, Czech Republic; mpolaskova@utb.cz; 3 Centre of Polymer Systems, Tomas Bata University in Zlin, Tr. T. Bati 5678, 760 01 Zlin, Czech Republic; stenicka@utb.cz; 4 Department of Animal Product Processing, Faculty of Food Technology, University of Agriculture in Krakow, Balicka 122, 30-149 Krakow, Poland; agnieszka.pluta-kubica@urk.edu.pl; 5 Department of Logistics, Faculty of Military Leadership, University of Defense, Kounicova 65, 662 10 Brno, Czech Republic; marketa.petova@unob.cz (M.P.); ondrej.novosad@unob.cz (O.N.)

**Keywords:** processed cheese, water addition, viscoelastic properties, textural properties, tribological properties, thermal properties

## Abstract

The current study investigated the impact of two-stage water addition on the selected properties of processed cheese (PC). In particular, the above-mentioned novel approach involved adding water in two stages during the PC manufacturing process. The effects of this process on the physicochemical, viscoelastic, textural, tribological, thermal, and organoleptic properties of PC were evaluated. For all examined PC samples, the elastic modulus consistently dominated over the viscous modulus (*G*′ > *G*″) across the entire frequency range. Moreover, it was observed that a smaller amount of initial water addition during the melting process resulted in a slight increase in the values of both viscoelastic moduli. The control sample exhibited the lowest lightness values, while it also showed the highest level of yellow coloring, suggesting that the two-stage addition of water affected the color of the PC samples. The results showed that the two-stage addition of water significantly influenced the physicochemical, viscoelastic, textural, tribological, thermal, and organoleptic properties of PC, leading to a modified texture, and thermal stability. Moreover, firmer PC products were obtained when a greater initial water level (first dosage; in the range of 60 to 90%) was utilized. This study could provide valuable information on the development of high-quality PC products with tailored functional properties, which can be important for the dairy industry.

## 1. Introduction

Processed cheese (PC) is a heat-treated dairy product and, from a physicochemical point of view, can also be characterized as a viscoelastic dairy-based gel, or can be defined as a dispersion of fat globules in a continuous protein network matrix [1]. PC manufacture is based on the heat treatment and mechanical stress of natural cheeses (of different types and varying degrees of maturity), and other dairy and non-dairy ingredients, including emulsifying salts (ESs), resulting in a smooth and homogeneous molten mass [2]. Thus, as several complex physicochemical phenomena (separation of calcium ions from the matrix of natural cheeses, protein hydration, and fat emulsification) are involved in the PC production process, new scientific studies are continuously added to deepen and develop knowledge in this field [3]. In general, PC can be produced from natural cheese, water, and ES, alongside a complex mixture of ingredients that are either of dairy or non-dairy origin. Water assumes a crucial role in determining the properties and functionality of PC, particularly influencing the textural and rheological characteristics and overall quality. Owing to its distinctive properties, water effectively dissolves the ES, hydrates proteins, and disperses other ingredients. The latter phenomenon could be attributed to water functioning as a low-viscosity lubricant within the protein matrix of PC, thereby improving elasticity and diminishing firmness. The relationship between water and the protein structure is critical; casein micelles interact with water, and it is suggested that a higher water content leads to a less firm protein matrix, resulting in higher values of meltability and spreadability. Furthermore, water is incorporated for economic reasons, chiefly to reduce costs [4].

Furthermore, two main approaches have been established to evaluate PC consistency. The first approach is rheological analysis, which examines changes in the sample under small shear deformation. Additionally, the second method involves the determination of the textural properties, which describes the behavior of the sample under large deformations. Rheology is used to study the deformation (primarily small) behavior or flow of fluids, and defines the relationship between the stress applied to a material and its subsequent deformation. Rheological analysis is crucial in food science and technology because it provides quantitative results on the mechanical properties of food products, allowing the consistency of the product to be characterized based on viscoelastic measurements [5]. The most important rheological properties of PC products are viscosity and elasticity, as most PCs belong to viscoelastic substances, thus presenting both elastic and viscous characteristics [6]. In addition, the texture profile analysis (TPA) method simulates the chewing process of food in the mouth. Based on this analysis, the key texture parameters of the product that are evaluated include hardness, cohesiveness, relative adhesiveness, chewiness, and gumminess [7]. Although these two methods exhibit significant correlation, rheological analysis is considered a more sensitive method compared to TPA [8]. Tribological analysis of food represents an innovative field of research. In the context of PC tribological measurements, the oral processing of food is simulated within the mouth. The method of the analysis mentioned above involves the interaction between two surfaces. In particular, during food consumption, one surface is represented by the tongue, while the other corresponds to the palate of the oral cavity. As a result, tribology can be described as the friction that food exhibits when compressed between two moving surfaces, specifically the tongue and the palate, when a thin layer of food is present. Therefore, this method provides valuable information on the friction (or lubrication) of food behavior during oral manipulation [9].

In addition, various PC forms and consistencies are available on the market, such as slices, shreds, spreads, and sauces [10]. Furthermore, the consistency of PC can be influenced by several factors [11], including the applied processing parameters (target temperature during manufacturing, stirring speed, holding time at the target temperature, and overall manufacturing time) and the possible application of various food additives as texture modifiers/enhancers, or the addition of water [12]. In addition to that, in the PC manufacturing process, water is incorporated to form a stable and smooth end product and to hydrate the applied ingredients [13].

Despite extensive research on PC formulation and processing, there remains a gap in the literature regarding the impact of two-stage water addition on PC techno-functional properties. In particular, water plays a crucial role in PC manufacturing, as it facilitates the hydration of the ingredients and contributes to the formation of a stable and homogeneous end product [13]. Although the behavior of water in dairy matrices has been studied to some extent, research on its role in PC remains limited. Most of the research conducted on water dynamics in cheese has focused on curd syneresis and water-holding capacity [14]. However, the scientific literature is missing a study characterizing two-stage water addition to PC or similar products.

Given the research gaps mentioned above, the current work aimed to evaluate the influence of the two-stage addition of water on the physicochemical, rheological, textural, tribological, thermal, and organoleptic properties of PC products. By elucidating these effects, this study could provide valuable information for optimizing PC production and improving product quality.

## 2. Materials and Methods

### 2.1. Ingredients Used for the Manufacture of the Processed Cheese Samples

The following ingredients were used in the manufacture of PC: Edam cheese blocks (Lacrum Velké Meziříčí s.r.o., Velké Meziříčí, Czech Republic; 7-week maturity) with a dry matter (DM) content of 30% (*w*/*w*) and a fat in dry matter (FDM) content of 50% (*w*/*w*); butter (Madeta a.s., České Budějovice, Czech Republic), with 82% *w*/*w* FDM, 85% *w*/*w* DM; water; and a mixture of ES (Fosfa a.s., Břeclav, Czech Republic), consisting of disodium hydrogenphosphate (Na_2_HPO_4_), sodium dihydrogen phosphate (NaH_2_PO_4)_, tetrasodium pyrophosphate (Na_4_P_2_O_7_), and sodium salt of polyphosphate, with the mean length n ≈ 20 (P20).

### 2.2. Manufacture of the Processed Cheese Samples

**Figure 1:**
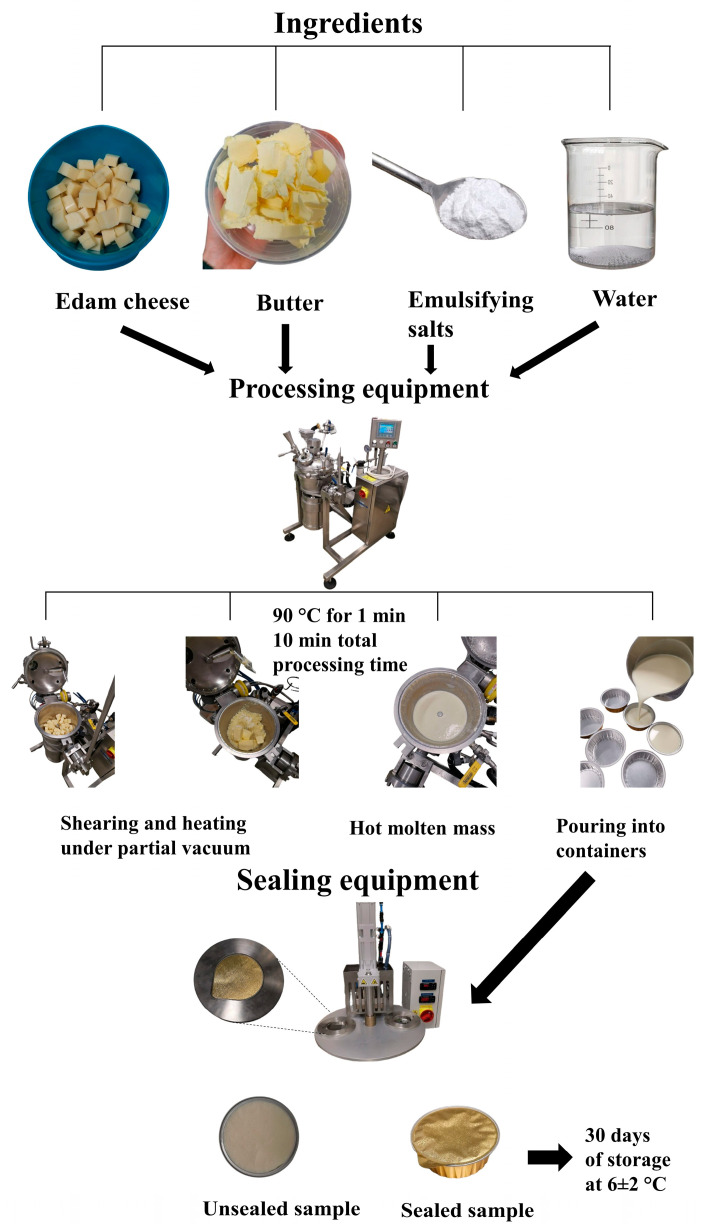
Schematic illustration of the basic ingredients and manufacturing steps utilized in the development of processed cheese products.

**Figure 2:**
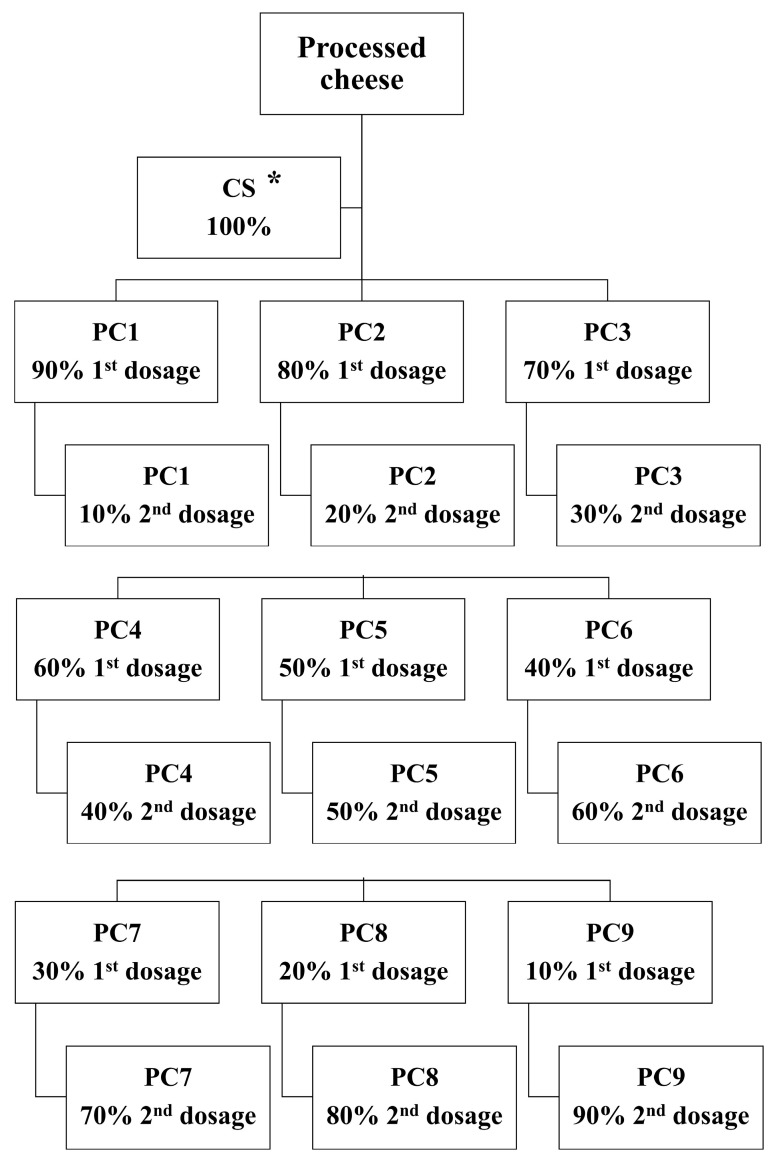
Schematic description of the experimental design with model processed cheese samples manufactured with the two-stage water addition process. * CS = Control sample; PC 1–9 samples of processed cheeses with different proportions of water content (1st dosage: 2nd dosage; PC 1 = 90:10; PC 2 = 80:20; PC 3 = 70:30; PC 4 = 60:40; PC 5 = 50:50; PC 6 = 40:60; PC 7 = 30:70; PC 8 = 20:80; PC 9 = 10:90% *w*/*w*).

The composition of the ingredients applied (Table 1) for PC samples was formulated to manufacture the final products with 45% (*w*/*w*) (DM) and 50% (*w*/*w*) fat (FDM) contents, respectively. Initially, Edam cheese was mechanically ground with NIROMIX (Nirosta s.r.o., Chlumec nad Cidlinou, Czech Republic) processing equipment (at 3000 rpm for 60 s). The remaining ingredients, including butter, ES (Fosfa a.s., Břeclav, Czech Republic), and water, were added to the apparatus (Figure 1). The mixture was stirred (at 3000 rpm) while indirect heating was applied simultaneously. The target processing temperature was set at 90 °C, with a holding time of 1 min. Overall, the total processing time was in the range of 8–10 min. In this study, a total of 10 PC samples were produced, and in 9 of them water was added in two steps. The principle of two-stage water addition during the processing involved dividing a specific water dosage into two parts. In particular, the first dosage of the water was added at the beginning along with the other ingredients, while the second dosage was added after 4 min of processing. For 9 PC samples, a two-stage water addition step was realized in different ratios (1st dosage–2nd dosage): 90:10, 80:20, 70:30, 60:40, 50:50, 40:60, 30:70, 20:80, and 10:90 (% *w*/*w*). A simple explanation of the 90:10 ratio is that 90% of the water was added at the beginning of the processing, and the remaining 10% was added during processing, specifically after 4 min of melting. The same principle was applied to the other water ratios until the final ratio of 10:90 was reached. The control sample (CS) was the only one manufactured without a two-stage water addition step. Figure 2 illustrates the schematic description of the experimental design with model PC samples manufactured with the two-stage water addition process. The final hot melt of each PC sample was poured into prepared aluminum containers (conical shape, with internal dimensions of 26.8 mm in height, 81.1 mm in diameter at the top, and 68.9 mm at the bottom) containing approximately 87 ± 5 g of the sample, and sealed with airtight aluminum lids with NovaSeal equipment (Nirosta s.r.o., Chlumec nad Cidlinou, Czech Republic). The PC samples were left to cool (25 ± 2 °C; approximately 6 h) and were then stored (6 ± 2 °C) until the analyses were performed. The PC samples were analyzed after 30 days of storage (at 6 ± 2 °C). The experiment was carried out 3 times, and 30 PC batches (10 PC samples × 3 repetitions = 30; n = 30) were manufactured.

### 2.3. Basic Physicochemical Analysis of the Processed Cheese Samples

The DM content of the PC samples was determined using the gravimetric method in accordance with ISO 5534:2004 [15] by drying the samples at a temperature of 102 ± 2 °C until a constant mass was achieved. The pH of PC samples was determined with a calibrated pH meter by inserting a glass-tipped electrode (Foodcare HI 99161, Hanna Instruments Czech s.r.o., Prague, Czech Republic) directly into the samples at three randomly selected locations. Furthermore, using the AquaLab 4TE device (Qi Analytical, s.r.o., Prague, Czech Republic, Decagon), the water activity (a_w_) of the PC samples was determined (25.0 ± 0.1 °C). A standard solution (a_w_ = 0.92 NaCl 2.33 molar in H_2_O; Qi Analytical, s.r.o., Prague, Czech Republic) was used before and during the measurements to ensure the precision of the results. All analyses were performed in nine replicates (three production batches with three repetitions; n = 9).

### 2.4. Determination of Processed Cheese Samples’ Viscoelastic Properties

A dynamic oscillatory shear rheometer (Thermo ScientificTM RheoStress 1; HAAKE Bremen, Germany) equipped with parallel plate geometry (35 mm in diameter) and a 1 mm gap was used to determine the viscoelastic properties of the PC samples. The measurements were conducted within the linear viscoelasticity region, with the shear stress amplitude set at 20 Pa during the frequency sweeps (0.1–100.0 Hz). Stress sweeps were performed between 1 and 100 Pa, at a frequency of 10 Hz and a temperature of 20 °C, to determine the linear viscoelasticity region. For every PC model sample, the viscoelastic characteristics were tested a minimum of three times (n = 3). The values of the elastic (*G*′) and viscous (*G*″) moduli were recorded during the analysis. Additionally, Equation (1) was used to compute the complex modulus (G*) values.

(1) $$G*\;=\;\sqrt{{\left(G\prime \right)}^{2}+{\left(G\prime \prime \right)}^{2}}$$

### 2.5. Texture Profile Analysis of the Processed Cheese Samples

The assessment of the textural characteristics of the PC was conducted using a TA.XT plus texture analyzer (Stable Micro Systems Ltd., Godalming, UK). Specifically, a texture profile analysis was carried out on the samples (20 ± 1 °C) to achieve a 20% deformation using a cylindrical probe (P20) with a diameter of 20 mm. Furthermore, the penetration rate was set at 2 mm·s^−1^, while the trigger force was maintained at 5 g. The PC container was placed under the probe for further compression. Every PC sample tested was measured on a minimum of six repetitions (n = 6). On the basis of the recorded force–time curves, the textural parameters of hardness, cohesiveness, and relative adhesiveness were subsequently evaluated [16].

### 2.6. Tribological Analysis of the Processed Cheese Samples

The principle of tribology lies in the study of friction, wear, and lubrication during the contact of two surfaces in relative motion [17]. Tribological measurements were conducted using a UMT TriboLab nanomechanical tribometer (Bruker s.r.o., Billerica, MA, USA) with a ball-on-plate geometry. To closely mimic the behavior of PC in contact with the tongue, a polyurethane textured surface platform was developed by mixing polyol (component A) and isocyanate (component B) (Table 2). Both components were thoroughly mixed at a temperature above 20 ± 2 °C. Subsequently, the two components were combined according to the prescribed ratio and mixed. The resulting blend was subsequently transferred into rectangular parallelepiped molds with dimensions of 80 mm in length, 45 mm in width, and 25 mm in thickness. The roughness was obtained by covering the bottom face of the mold (80 × 45 mm) with sandpaper (K100; OBI Group Sourcing GmbH, Wermelskirchen, Germany) with a grain size class of P100 (defined by the Federation of European Producers of Abrasives). The class indicates the number of meshes per square inch of sieve surface for grain sizing: a greater class number means a finer grain size and a smoother surface. Finally, demolding took place after 24 h (23 ± 2 °C) [18].

Before each tribological measurement, a thin, uniformly spread, PC layer, approximately 30 × 30 × 2 mm in size, was applied to the developed platform. The platform with the tested sample on it was then placed on the lower, movable module of the tribometer. Subsequently, an 8 mm stainless-steel ball was pressed into the PC sample layer with a defined axial force of 2 N. Once the axial force stabilized, a cyclic motion was initiated, generating sliding friction in a linear relative movement (frequency of 0.1 Hz and path of 10 mm). Throughout the process, the tangential frictional force was measured, which was used to calculate the coefficient of friction (CoF). The CoF refers to the ratio of the friction force to the normal force, which is defined as Equation (2):

(2) $$\mu =\frac{dF}{dN}$$

where F represents the torque (Nm) and N is equivalent to 1 N [17]. All measurements were performed at least 6 times (n = 6).

### 2.7. Differential Scanning Calorimetry of the Processed Cheese Samples

Differential scanning calorimetry (DSC) analysis was performed with a DSC 250 Discovery device (TA Instruments, New Castle, DE, USA) with Tzero technology (T1) under laboratory conditions. In the first phase of the experiment, 10.0 ± 1.1 mg of PC samples were weighed in hermetically sealed pans, and an empty pan was used as a reference. The samples were then pressed with a lid featuring a 1 mm pin. Measurements were carried out in a nitrogen atmosphere (50 mL^−1^) with a cooling rate of 10 °C min^−1^, within a temperature range of 25 °C to −50 °C, followed by an isothermal step at −50 °C for 1 min. Heating was recorded at a rate of 5 °C^−1^ over a temperature range from −50 °C to 80 °C. The water status was evaluated for all PC samples using the ice melting temperature and the relevant enthalpy of fusion ∆H_fus_ [19]. The crystallization peak (T_cryst_) and the freezing enthalpy ∆H_cryst_ were evaluated for the cooling cycle of all PC samples. For the heating cycle, endothermal onset (T_onset_), melting peak temperature (T_p,m_), and ∆H_fus_ were rated for each thermogram. The corresponding enthalpies of these processes were calculated by integrating the area under the thermogram and expressed as a specific enthalpy in J·g^−1^ (normalized enthalpy). The specific enthalpy at constant pressure corresponds to the system’s internal energy, i.e., the binding forces in the sample. This is related to the number of bonds required to maintain the native (complex) conformation of the sample [20]. The content of freezing water (i.e., freezing free and freezing bound water) in PC was determined using Equation (3) as the ratio of the sample’s ΔH_fus_ to the enthalpic change in pure water (307.5 J·g^−1^) measured under identical conditions, rather than the standardized heat of fusion value (ΔH^0^_m,H₂O_ = 333.5 J·g^−1^) for pure water [17].

(3) $$W_{f,s}\;(\%)=\frac{{\Delta H}_{fus}}{{\Delta H}_{m,H2O}^{0}}\times 100$$

where W_f,s_ is the freezing free and the freezing bound water content [21].

### 2.8. Instrumental Determination of the Color of the Processed Cheese Samples

The instrumental color properties of the PC samples were analyzed using an UltraScan PRO spectrophotometer (Hunter Associates Laboratory, Inc., Reston, VA, USA). The evaluation was based on the CIE Lab color scale (L*a*b*) in reflectance mode, excluding specular reflection, with illuminant D65 (average daylight) and an observer angle of 10°. In addition, the L* parameter, representing lightness, was measured within a scale ranging from 0 to 100 (where 0 corresponds to black and 100 to white). The a* parameter indicated the position on the red–green axis (with −a* values corresponding with green and +a* values corresponding with red). Similarly, the parameter b* reflected the yellow–blue axis (with −b* values indicating blue and +b* values indicating yellow) [22]. All PC samples were measured at least 9 times (3 manufactured batches × 3 repetitions; n = 9).

The hue angle (*h*°) defines the degree of the dominant spectral component, such as red, green, or blue, within a range of 0° to 360°. Specifically, an angle of 0° or 360° represents a red hue, while angles of 90°, 180°, and 270° correspond to yellow, green, and blue hues, respectively. In general, the combination of a*, b*, and h°* provides a more detailed description of the color and is determined using the following Equation (4):

*h*° = *tan*^−1^(*a*^∗^/*b*^∗^) (4)

The chroma (C*) parameter represents the intensity or saturation of a color and is defined by Equation (5):

(5) $$C^{*}={{(a}^{*2}+b^{*2})}^{0.5}$$

Furthermore, the whiteness index (*WI*) of the PC samples was determined using the following Equation (6):

(6) $$WI={\left[{\left(100-L^{*}\right)}^{2}+a^{*2}+b^{*2}\right]}^{0.5}$$

Additionally, color changes were assessed as the total color difference (∆*E*12), which quantifies the extent of color variation between two samples using the following Equation (7):

(7) $$∆E12*={\left[{\left(∆L^{*}\right)}^{2}+{\left(∆a^{*}\right)}^{2}+{\left(∆b^{*}\right)}^{2}\right]}^{0.5}$$

### 2.9. Sensory Analysis

The PC sensory analysis was performed at the Department of Food Technology, Faculty of Technology, Tomas Bata University, in Zlin (Czech Republic) according to ISO 8586 [23]. Table 3 presents the attributes of the sensory evaluators. PC samples were labeled with four-digit codes and randomly served on white plates. To avoid carryover effects, water and white bread were offered during the testing and evaluation of the PC samples. According to ISO 8589 [24], sensory analysis was performed in sensory booths at a controlled temperature of 22 ± 2 °C and with standard lighting (daylight-type bulbs were used). Furthermore, the assessors were asked to evaluate PC samples based on appearance, consistency, hardness, spreadability, flavor, and off-flavor attributes. For the evaluation, 7-point hedonic scales (1: excellent, 3: good, 5: less good, 7: unacceptable) were used for appearance, consistency, hardness, flavor, and spreadability, and off-flavors (1: negligible, 4: medium, and 7: excessive). Each level on the 7-point scale was described using specific intensity terms [17].

### 2.10. Statistical Analysis

The Shapiro–Wilk test was employed to assess the normal distribution of the obtained data (significance level set at 0.05; using Minitab 16, Minitab Ltd., Coventry, UK). For small datasets, ISO 5479 [25] recommends the Shapiro–Wilk test as a suitable method for evaluating normal distribution. The employment of parametric tests was dismissed due to the unacceptable normal distribution of the data (*p* < 0.05). Consequently, a nonparametric analysis was conducted using the Kruskal–Wallis and Wilcoxon tests for variance (Minitab 16 software; Minitab Ltd., Coventry, UK), maintaining a significance level of 0.05.

## 3. Results and Discussion

### 3.1. Basic Physicochemical Analysis and Water Activity of the Processed Cheese Samples

The results of the basic physicochemical analysis of the PC samples are summarized in Table 4. The results of the DM content ranged from 44.82% to 46.58% (*w*/*w*) for all samples tested (*p* > 0.05). Furthermore, pH is a parameter that can significantly influence the physicochemical properties and overall quality of PC [26,27,28]. According to a study by Lee et al. [6], the optimal pH value for PC with spreadable consistency ranges from 5.5 to 5.8. In general, it can be concluded that PC samples presented pH values that could be characterized as acceptable (*p* > 0.05). The resulting values of a_w_ for the individual PC samples, as presented in Table 4, exhibited minimal variation. In particular, the range of a_w_ values was between 0.976 and 0.978 (*p* > 0.05). According to Glass and Doyle [29], typical PC a_w_ values are in the range of 0.910–0.960, effectively inhibiting the growth of certain bacteria. Furthermore, the latter authors reported that PC is considered a safe food product due to its physicochemical properties, manufacturing technology, and appropriate storage conditions. On the basis of the data obtained, it can be concluded that the two-stage water addition process did not affect the resulting values of the basic physicochemical analysis and a_w_ results.

### 3.2. Viscoelastic Properties of the Processed Cheese Samples

**Figure 3:**
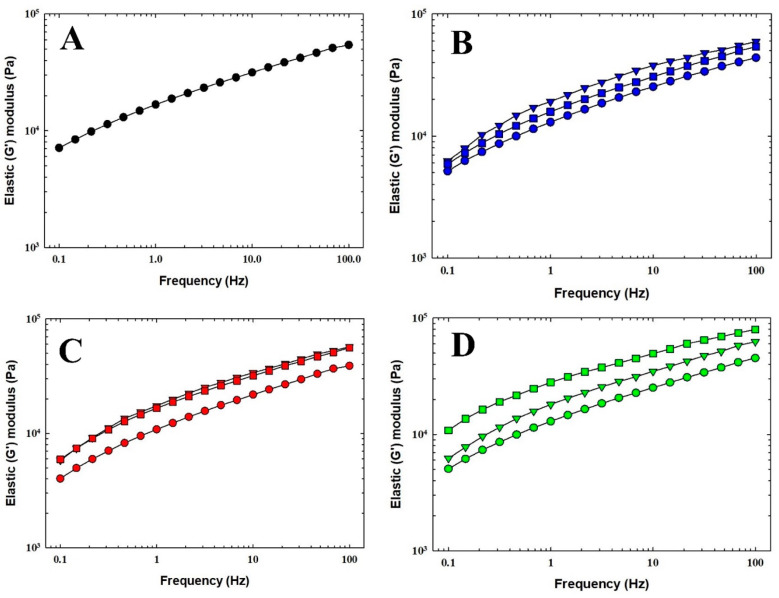
The dependence of elastic (*G*′) modulus of the processed cheese samples manufactured with two-stage water addition on frequency (0.1–100.0) after 30 days of storage (6 ± 2 °C; n = 6). The control sample (manufactured without the two-stage water addition step) is also included (**A**). PC 1—*blue circle*, PC 2—*blue triangle*, and PC 3—*blue square* (**B**); PC 4—*red circle*, PC 5—*red triangle*, and PC 6—*red square* (**C**); PC 7—*green circle*, PC 8—*green triangle*, and PC 9—*green square* (**D**). PC 1—90:10; 1st dosage–2nd dosage; PC 2—80:20; 1st dosage–2nd dosage; PC 3—70:30; 1st dosage–2nd dosage; PC 4—60:40; 1st dosage–2nd dosage; PC 5—50:50; 1st dosage–2nd dosage; PC 6—60:40; 1st dosage–2nd dosage; PC 7—70:30; 1st dosage–2nd dosage; PC 8—80:20; 1st dosage–2nd dosage; PC 9—90:10; 1st dosage–2nd dosage; % *w*/*w*. Samples of processed cheese were evaluated after 30 days of storage at 6 ± 2 °C (n = 3).

**Figure 4:**
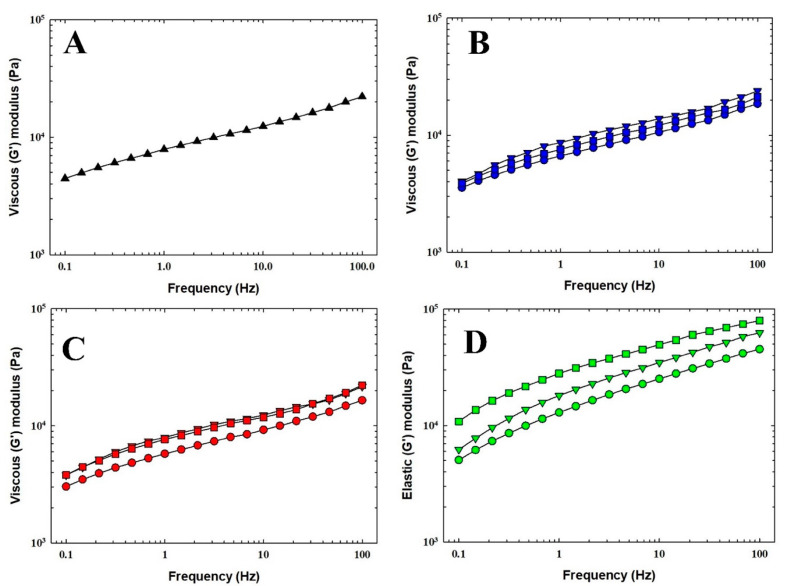
The dependence of the viscous (*G″*) modulus of the processed cheese samples manufactured with two-stage water addition on frequency (0.1–100.0) after 30 days of storage (6 ± 2 °C; n = 6). The control sample (manufactured without the two-stage water addition step) is also included (**A**). PC 1—*blue circle*, PC 2—*blue triangle*, and PC 3—*blue square* (**B**); PC 4—*red circle*, PC 5—*red triangle*, and PC 6—*red square* (**C**); PC 7—*green circle*, PC 8—*green triangle*, and PC 9—*green square* (**D**). PC 1—90:10; 1st dosage–2nd dosage; PC 2—80:20; 1st dosage–2nd dosage; PC 3—70:30; 1st dosage–2nd dosage; PC 4—60:40; 1st dosage–2nd dosage; PC 5—50:50; 1st dosage–2nd dosage; PC 6—60:40; 1st dosage–2nd dosage; PC 7—70:30; 1st dosage–2nd dosage; PC 8—80:20; 1st dosage–2nd dosage; PC 9—90:10; 1st dosage–2nd dosage; % *w*/*w*. Samples of processed cheese were evaluated after 30 days of storage at 6 ± 2 °C (n = 3).

Rheological analysis is a fundamental method in the food industry, as it provides valuable information on the mechanical properties of food. Additionally, the consistency of PC can be effectively evaluated by determining its viscoelastic properties [5]. Figure 3 and Figure 4 illustrate the development of *G*′ and *G*″ moduli values as a function of frequency for selected PC samples. For all tested PC samples, the elastic modulus consistently dominated over the viscous component (*G*′ > *G*″) across both low- and high-frequency ranges. Moreover, a smaller amount of initial water addition during the melting process resulted in a slight increase in the values of both viscoelastic moduli. In particular, the viscoelastic character of PC is predominantly influenced by the properties of the primary component that forms the protein network [6]. Furthermore, Winter and Chambon [30] reported that higher values of *G*′ and *G*″, (and even G*) indicate increased product stiffness. It could be stated that a lower initial water addition, compared to water introduced later during the melting process, led to a marginal increase in the stiffness of the final PC samples.

The viscoelastic properties of model processed cheese samples were also determined using the phase shift angle *tan δ* and G*. A reference frequency of 1 Hz was selected for a better comparison. The values of G* and *tan δ* are shown in Table 4. The resulting values of G* varied slightly between PC samples and did not show a consistent trend. Nevertheless, based on the results, it can be concluded that the highest G* values were observed in PC 7 and PC 9. On the contrary, as determined by its viscoelastic properties, the least firm sample was identified as sample PC 1. Only minor differences were observed in the remaining PC samples. Several authors, such as Lee et al. [31] or Sołowiej et al. [32], have studied the rheological properties of PC. However, no studies have specifically investigated the effects of the two-stage addition of water during the melting process.

Furthermore, Table 4 presents the *tan δ* values, a parameter that reflects the degree of viscoelasticity in the PC samples. Dimitreli and Thomareis [33] reported that materials with a *tan δ* value of 1 exhibit a balanced behavior, acting equally as a solid and a liquid material. Conversely, materials with *tan δ* < 1 demonstrate predominantly elastic properties, while those with *tan δ* > 1 are characterized by a more viscous nature [33]. The *tan δ* values for the PC samples (Table 4), at a reference frequency of 1 Hz, were consistently below 1, suggesting that the PC samples mainly demonstrated a more elastic character.

### 3.3. Texture Profile Analysis of the Processed Cheese Samples

The results of hardness development with respect to the use of a two-stage water addition process during PC production are presented in Table 4. The results show that the two-stage addition of water affected the hardness values of the PC samples. In particular, the PC with the lowest hardness was sample PC 1, where 90% of the water was added at the beginning of the melting process, and the remaining 10% was added after 4 min of melting. However, PC 9 demonstrated a higher value of hardness than the previously mentioned sample. Therefore, it can be concluded that water plays an important role in the production process and contributes to reducing the hardness of the product. This assertion aligns with the findings of Dimitreli et al. [33], who highlighted the decreased formation of protein matrices due to the influence of water and moisture. The lower hardness values can also be attributed to a shift in the system’s pH. Specifically, for PC with a relatively higher pH level (6.5–6.7), one can anticipate a reduction in the strength of electrostatic interactions. As a result, this weakens the gel, producing a product with an excessively soft consistency. Additionally, when the pH of PC is reduced to around 5.5 to 5.2, near to the isoelectric point of the caseins, it leads to the formation of a much firmer product that might have a crumbly texture [34].

The following texture parameters evaluated were elasticity, which is determined by the rate of return of the deformed material to its original shape; cohesiveness (the strength of the internal bonds in the processed cheese samples); and relative adhesiveness (the force required to remove the adhering substance from a material) [6]. The resulting elasticity values are given in Table 4. Based on the obtained values, it is evident that there were no significant differences between the PC samples. For elasticity, relative stickiness, and cohesiveness, the gradual addition of water during the manufacture of processed cheese had no demonstrable effect.

### 3.4. Tribological Analysis of the Processed Cheese Samples

**Figure 5:**
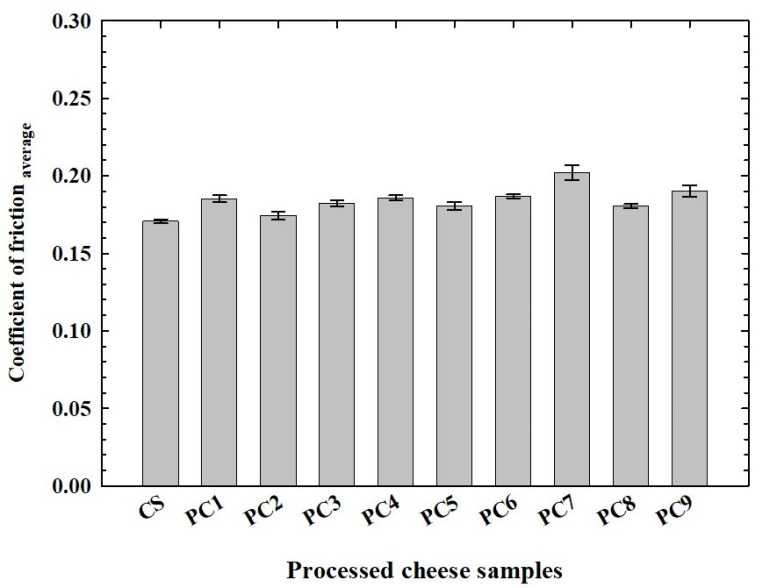
Coefficient of friction values of processed cheese samples manufactured with two-stage water addition. The control sample (**CS**; manufactured without the two-stage water addition step) is also included. PC 1—90:10; 1st dosage–2nd dosage; PC 2—80:20; 1st dosage–2nd dosage; PC 3—70:30; 1st dosage–2nd dosage; PC 4—60:40; 1st dosage–2nd dosage; PC 5—50:50; 1st dosage–2nd dosage; PC 6—60:40; 1st dosage–2nd dosage; PC 7—70:30; 1st dosage–2nd dosage; PC 8—80:20; 1st dosage–2nd dosage; PC 9—90:10; 1st dosage–2nd dosage; % *w*/*w* [n = 3; the results are expressed as means (columns) and standard deviations (bars)].

The results of the tribological analysis for all measurements of the individual PC samples are presented in Figure 5. It can be concluded that the PC 3 sample exhibited the highest values of CoF. However, based on the results, it can be concluded that the lubrication properties of the PC 7 sample were governed by higher interactions between the structure units in the network, as higher degrees of interaction resulted in a higher CoF. A similar trend was observed during the determination of viscoelastic properties, further confirming that these two methods correlate with each other, as was also previously stated by Ningtyas et al. [35]. However, no significant differences were found between the other PC samples tested. Therefore, the two-stage water addition step used in PC production had no impact on the tribological properties of the samples. In summary, a deeper understanding of the tribological properties of PC could allow manufacturers to refine both the formulation of the ingredients and the production process. As a result, PC products could be designed to effectively satisfy consumer expectations regarding both flavor and mouthfeel.

### 3.5. DSC of the Processed Cheese Samples

**Figure 6:**
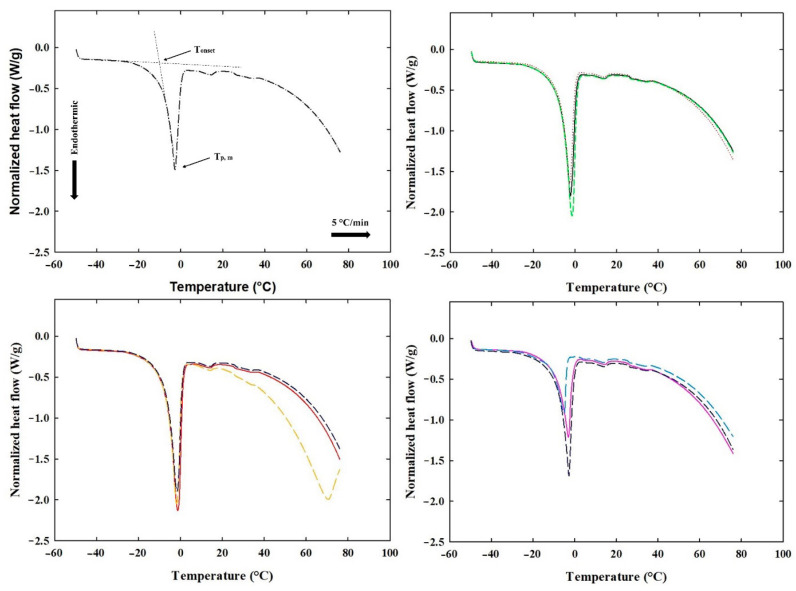
Differential scanning calorimetry patterns exhibiting the endothermic peak of ice melting for processed cheese samples manufactured with two-stage water addition. Processed cheeses were sampled after 30 days of storage at 6 ± 2 °C (n = 6). The control sample (manufactured without a two-stage water addition step) is also included (part A; *black dashed-dotted line*). PC 1 (Part B; *black solid line*)—90:10; 1st dosage–2nd dosage; PC 2 (Part B; *red dotted line*)—80:20; 1st dosage–2nd dosage; PC 3 (Part B; *green dashed line*)—70:30; 1st dosage–2nd dosage; PC 4 (Part C; *red solid line*)—60:40; 1st dosage–2nd dosage; PC 5 (Part C; *yellow dashed line*)—50:50; 1st dosage–2nd dosage; PC 6 (Part C; *blue dashed line*)—60:40; 1st dosage–2nd dosage; PC 7 (Part D; *purple solid line*)—70:30; 1st dosage–2nd dosage; PC 8 (Part D; *light blue dashed line*)—80:20; 1st dosage–2nd dosage; PC 9 (Part D; *dark blue dashed line*)—90:10; 1st dosage–2nd dosage; *w*/*w*. Processed cheeses were sampled after 30 d of storage at 6 ± 2 °C (n = 3).

DSC analysis readily evaluates the amount of freezable water and water molecules that cannot crystallize as a result of specific interactions within the studied matrix. The quantity of water that freezes during food cooling can be determined from the enthalpy of ice melting during the cooling cycle [13]. As the concentration of low-molecular-weight compounds such as carbohydrates and salts increases, the freezing point decreases, which is one of the colligative properties [36]. The DSC thermograms illustrate the crystallization behavior (cooling) of both the bound and free water present in the sample. During the interaction between water, salts, and biopolymer molecules (such as proteins and lipids), three forms of water can be observed: non-freezing water, freezing bound water, and free water. Specifically, non-freezing bound water is closely associated with the polymeric matrix and does not display a phase transition detectable by calorimetric analysis. On the contrary, freezing bound water is less tightly bound to the matrix and exhibits melting and crystallization temperatures that differ significantly from the crystallization temperature of bulk water. Lastly, free water presents melting and crystallization values similar to those of bulk water [37]. During DSC analysis, the influence of two-stage water addition on the content of freezable free and bound water based on ∆H_fus_ in PC samples was examined. Figure 6 presents the relationship between heat flow and temperature for the various PC samples. The DSC curve displays a sharp crystallization peak, indicating a very rapid crystallization process, during which a significant amount of energy was released in a relatively short time frame. Specifically, the water present in the sample began to crystallize at temperatures ranging from −14 °C to −21 °C, depending on the ratios of water added at the beginning of the experiment and at the 4 min mark. From the results, it is evident (as shown in Figure 6) that the two-stage addition of water affected the binding of water to the structure of PC. The endothermic peak (heating) observed at temperatures ranging from T_onset_ = −6 °C to −11 °C represented the melting point of frozen water bound within the PC samples. Furthermore, the melting peak of water at temperatures lower than 0 °C can be attributed to the melting of free frozen water, known as “freezing point depression”, which is typically lower than that of bulk free water due to the presence of melting salts in the sample. According to the study by Clausse [38], the formation of ice at temperatures lower than 0 °C is linked to the process of supercooling, which is characteristic of water/oil emulsions. In this study, PC samples exhibited supercooling, causing ice crystallization to shift to subzero temperatures. Gliguem et al. [13] mainly attributed this phenomenon to cheese samples relying on hydrophilic constituents and the water being retained within the microdomains created by fat droplets.

The results of the DSC measurement (the cooling cycle T_cryst_, ΔH_cryst_; and the heating cycle T_onset_, T_p,m_, and ΔH_fus_) for PC samples produced with a two-stage water addition are presented in Table 5. Furthermore, the W_fs_ values were determined using Equation (3), representing the content of freezable free and freezable bound water. The results showed that two-stage water addition influenced the binding of water to the structure of PC samples. Moreover, the heating curve further confirmed that sample PC 2 exhibited the most tightly bound water within the sample (Table 5). Thus, it can be concluded that sample PC 2 appeared to be the most favorable for preparation in terms of its stability and organoleptic properties. This is primarily due to the lowest amount of free water present in this sample. However, according to a study by Van der Sman and Boer [39], it should be stated that the literature is not unambiguous in terms of bound water. Water that remains unfrozen at temperatures below the equilibrium freezing point, even in the presence of ice, is referred as “bound” water, as described by Wolfe et al. [40]. Additionally, research by Gliguem et al. [13] has demonstrated that certain food materials can undergo a glass transition, causing a portion of the bulk water to remain unfrozen by transitioning to an amorphous glassy state rather than crystallizing into ice [39,40]. Due to the frequent occurrence of these different behaviors, analyzing the state of the water in PC is inherently a complex phenomenon.

### 3.6. Instrumental Color Analysis of the Processed Cheese Samples

Table 4 presents the results obtained from the instrumental color evaluation. For evaluation, the values of lightness *L*, chromaticity a*, and chromaticity b* were first determined. As indicated by the L* values presented in Table 4, all samples fell within the range of 90.83–91.77, suggesting a high degree of lightness, which aligns with the findings of Kristensen et al. [41]. Consequently, all the samples were identifiable as a pale-yellow color with a noticeable greenish tint. This observation is further supported by the negative a* values recorded for all PC samples, which, in turn, signified the presence of a green tint. The CS exhibited the lowest lightness, while also showing the highest level of yellowness. This suggests a clear distinction between the samples, indicating that the two-stage addition of water influenced the color of the PC samples.

The C* values in Table 4 represent the chroma for the PC samples. Compared to the CS, which has a C* value of 15.50 ± 0.64, all other samples exhibit lower chroma values, ranging from 12.54 to 13.64. This indicates that, in relation to the CS, the other samples presented a lower color saturation. Moreover, despite variations in water content between the samples, these differences lead to only minimal changes in color saturation, suggesting that the overall intensity of the color was not significantly affected.

The Δ*E*12 values presented in Table 4 represent the total color difference between the samples, quantifying the perceptibility of color changes. The Δ*E*12 value is not specified for the CS, as it serves as a reference point. In contrast, the Δ*E*12 values for PC samples range from 1.94 to 3.08, with PC 1 exhibiting the lowest value (1.94 ± 0.47) and PC 7 showing the highest value (3.08 ± 0.50). These results indicate noticeable color differences between the CS and the PC samples. Furthermore, it can be observed that as the water content in the samples varies, the color difference tends to increase slightly. However, the overall changes in color remain relatively small, as all Δ*E*12 values are below three units. PC 7 shows the highest value (3.08 ± 0.50). Particularly, PC 7 has a Δ*E*12 value exceeding 3, which indicates a perceptible color difference that may be noticeable to the human eye. This suggests that, in the case of PC 7, the color shift is more significant compared to the other samples. This suggests that, while two-stage water addition has some effect on color, the color shifts are moderate and do not represent significant alterations. Moreover, it is important to note that if Δ*E*12 is less than 1, differences in color are not noticeable to the human eye. Specifically, when 1 < Δ*E*12 < 3, small color differences may be discernible to the human eye, and when Δ*E*12 exceeds value 3, color differences become more noticeable to the observer [42].

The whiteness index (WI) values depicted in Table 4 quantify the degree of whiteness of the PC samples. The *WI* values for the CS and the PC samples range from 81.99 to 84.94. Specifically, the CS has a *WI* value of 81.99 ± 0.34, while the highest value is observed in PC 7 (84.94 ± 0.11). These values suggest that the PC samples exhibit a relatively high degree of whiteness, with some variation between the different samples. Furthermore, the *WI* values show a general trend of increasing whiteness as the proportion of water in the samples increases. This indicates that a higher water content may have a slight whitening effect on the PC. However, the differences in *WI* values are not particularly large, suggesting that the effect of water content on whiteness is relatively subtle. Overall, the whiteness index values indicate that while the PC samples show slight differences in whiteness, the overall level of whiteness remains quite similar across all samples.

Dairy products typically exhibit a white to slightly yellowish color, a characteristic that is mainly due to the presence of milk fat and naturally occurring pigments, including retinol and carotenoids [41,43]. In general, color is an important sensory attribute of cheese, reflecting its ripeness and freshness. Factors such as milk composition, treatments, ripening techniques, food additives, and microflora activity significantly influence its color [44]. Therefore, color evaluation provides a quick and effective method of detecting anomalies or defects in cheese production. Additionally, the color and visual appearance of food play a crucial role in shaping consumer expectations, which, in turn, influence their preferences and the identification of food items [45].

### 3.7. Sensory Analysis

Sensory characteristics of PC (appearance, consistency, hardness, spreadability, flavor, and off-flavor) manufactured with two-stage water addition are depicted in Table 6. The evaluation aimed to determine whether the assessors could detect differences between PC samples produced with the two-stage water addition manufacturing technique. All PC samples received positive ratings for their appearance, flavor, and off-flavor attributes. All PC samples exhibited excellent creamy and cheesy flavors, entirely free of any off-flavors. The samples that received the highest ratings for consistency were PC 5 (50% of the water was added at the beginning of the melting process, and the remaining 50% after 4 min of melting), which showed excellent consistency, being slightly harder. Based on a study by Gliguem et al. [13], it can be concluded that the addition of water in two stages can have an effect on the consistency of the resultant PC. This is because water facilitates the dissolution of calcium-chelating salts, hydrates proteins, and aids in dispersing components. Additionally, casein is better incorporated into the melt and is more effectively influenced by melting salts [46,47]. Other samples, where more water was added at the beginning of the melting process and less was added during it, were also evaluated to have better consistency. Moreover, the hardness of the PC was also evaluated, and it was found that the samples PC 7 and PC 9 exhibited the highest hardness values. In the case of spreadability, sample PC 7 was rated the worst, as it was not spreadable at all. Regarding the other samples, none exhibited optimal spreadability. Only samples CS, PC 1, PC 2, and PC 5 showed some degree of spreadability, although this was of poorer quality. The reasons for reduced spreadability or consistency may vary, including an inappropriate choice of raw materials (e.g., the maturity of natural cheese), processing parameters, or storage conditions [26].

## 4. Conclusions

This study evaluated the impact of the two-stage addition of water on selected properties (physicochemical, viscoelastic, textural, tribological, thermal, and organoleptic) of processed cheese. The two-stage water addition process involves adding water during PC manufacturing in two separate stages. The results showed that the two-stage water addition process influenced rheological behavior, textural characteristics, tribological properties, thermal stability, and organoleptic characteristics. Specifically, the two-stage process influenced PC textural properties, while the friction coefficient was reduced and the thermal stability improved. Moreover, the process enhanced the organoleptic properties of the PC, such as its flavor, aroma, and overall acceptability. This research could offer significant insights into the effects of two-stage water addition on processed cheese, and can be used to optimize the manufacturing process, improve product quality, and enhance consumer satisfaction. Furthermore, a practical benefit derived from this study is the ability to tailor the consistency of processed cheese according to consumer preferences. For consumers who favor processed cheese with a softer consistency, a reduced initial water addition (first stage; up to 50%) can be employed. Conversely, for those desiring a firmer product, a greater initial water addition (first stage; ranging from 60% to 90%) is feasible during the melting process.

## Figures and Tables

**Table 1:** Raw material formulation of processed cheese samples manufactured with two-stage water addition and with the following target values: 45% *w*/*w* DM content and 50% *w*/*w* fat in DM content.

Raw Materials	Ingredient Composition (% *w*/*w*)									
	CS *	PC 1	PC 2	PC 3	PC 4	PC 5	PC 6	PC 7	PC 8	PC 9
Edam cheese ^1^	50.60	50.60	50.60	50.60	50.60	50.60	50.60	50.60	50.60	50.60
Butter	13.70	13.70	13.70	13.70	13.70	13.70	13.70	13.70	13.70	13.70
Water addition	33.40									
First stage		30.06	26.72	23.38	20.04	16.70	13.36	10.02	6.68	3.34
Second stage		3.34	6.68	10.02	13.36	16.70	20.04	23.38	26.72	30.06
Amount of emulsifying salt	2.30	2.30	2.30	2.30	2.30	2.30	2.30	2.30	2.30	2.30
Na_2_HPO_4_	39.00	39.00	39.00	39.00	39.00	39.00	39.00	39.00	39.00	39.00
NaH_2_PO_4_	18.00	18.00	18.00	18.00	18.00	18.00	18.00	18.00	18.00	18.00
Na_4_P2O_7_	21.00	21.00	21.00	21.00	21.00	21.00	21.00	21.00	21.00	21.00
P20	22.00	22.00	22.00	22.00	22.00	22.00	22.00	22.00	22.00	22.00

^1^ Dutch-type semi-hard cheese, 7-week maturity. * CS = Control sample; PC 1–9 samples of processed cheeses with different proportions of water content (1st dosage: 2nd dosage; PC 1 = 90:10; PC 2 = 80:20; PC 3 = 70:30; PC 4 = 60:40; PC 5 = 50:50; PC 6 = 40:60; PC 7 = 30:70; PC 8 = 20:80; PC 9 = 10:90% *w*/*w*).

**Table 2:** Physical properties of the material for the manufacture of patterned surface pads (Axson technologies data sheet).

Physical Properties	Component A	Component B	Mixture
Composition	UR 58300 POLYOL	UR 5803 ISO	
Mixing ratio (by weight)	100	10	
State	Liquid	Liquid	Liquid
Color	Beige	Amber	Beige
Viscosity (mPa.s)	6900	2000	4000
Density (kg/L)	1.37	1.16	1.35
Processing time (25 °C)			15–20 min

**Table 3:** Characteristics of participants at sensory analysis of processed cheese samples.

Experiment	Panelist	*n*	Gender (%)		Age (Years)		
			Female	Male	Total	Female	Male
PC samples	Experienced	4	50	50	44.00 ± 4.24	43.50 ± 0.71	44.50 ± 7.78
	Trained	8	75	25	32.67 ± 6.31	28.33 ± 4.13	37.00 ± 8.49

**Table 4:** Analysis results for processed cheese samples manufactured with two-stage water addition after 30 days of storage (6 ± 1 °C; results are expressed as mean ± SD).

Parameter	Processed Cheese									
	CS *	PC 1	PC 2	PC 3	PC 4	PC 5	PC 6	**PC 7**	**PC 8**	**PC 9**
DM content (% *w*/*w*)	46.22 ± 1.30 a	44.82 ± 0.84 a	45.22 ± 1.01 a	45.59 ± 1.48 a	45.54 ± 0.98 a	46.22 ± 0.84 a	45.88 ± 1.00 a	46.03 ± 0.60 a	46.58 ± 0.68 a	45.49 ± 1.03 a
Fat content (% *w*/*w*)	19.98	19.78	19.88	19.75	19.81	19.92	19.91	19.88	19.89	19.95
pH (-)	5.51 ± 0.01 a	5.51 ± 0.04 a	5.48 ± 0.01 a	5.46 ± 0.02 a	5.52 ± 0.03 a	5.52 ± 0.05 a	5.45 ± 0.02 a	5.56 ± 0.05 a	5.47 ± 0.01 a	5.59 ± 0.03 a
Water activity (a_w_) (-)	0.975 ± 0.01 a	0.975 ± 0.01 a	0.977 ± 0.01 a	0.977 ± 0.01 a	0.976 ± 0.01 a	0.977 ± 0.01 a	0.978 ± 0.01 a	0.978 ± 0.01 a	0.978 ± 0.01 a	0.978 ± 0.02 a
Lightness (L*)	90.83 ± 0.34 a	91.39 ± 0.17 b	91.60 ± 0.20 b	91.69 ± 0.02 b	91.65 ± 0.22 b	91.49 ± 0.28 b	91.50 ± 0.10 b	91.66 ± 0.11 b	91.77 ± 0.12 b	91.73 ± 0.19 b
Chromaticity on a green-to-red axis (a*)	−0.44 ± 0.01 a	−0.48 ± 0.03 b	−0.52 ± 0.07 c	−0.45 ± 0.02 a	−0.47 ± 0.03 b	−0.40 ± 0.15 d	−0.49 ± 0.02 b	−0.45 ± 0.03 a	−0.39 ± 0.06 a	−0.45 ± 0.01 b
Chromaticity on a blue-to-yellow axis (b*)	15.50 ± 0.64 a	13.63 ± 0.21 b	12.78 ± 0.04 b	12.92 ± 0.13 b	12.73 ± 0.28 b	12.69 ± 0.17 b	13.20 ± 0.52 a	12.53 ± 0.21 b	13.04 ± 0.17 a	13.14 ± 0.04 a
Hue angle (h*; °)	−1.54 ± 0.02 a	−1.54 ± 0.03 a	−1.53 ± 0.01 a	−1.54 ± 0.01 a	−1.53 ± 0.02 a	−1.54 ± 0.01 a	−1.54 ± 0.01 a	−1.54 ± 0.01 a	−1.54 ± 0.01 a	−1.54 ± 0.01 a
Chroma (C*)	15.50 ± 0.64 a	13.64 ± 0.21 b	12.79 ± 0.04 c	12.93 ± 0.13 c	12.74 ± 0.28 c	12.70 ± 0.17 c	13.21 ± 0.52 b	12.54 ± 0.21 c	13.05 ± 0.17 b	13.15 ± 0.04 b
Total color difference (∆*E*12)	-	1.94 ± 0.47 a	2.82 ± 0.63 b	2.72 ± 0.61 b	2.88 ± 0.39 b	2.88 ± 0.50 b	2.39 ± 0.28 b	3.08 ± 0.50 c	2.63 ± 0.53 b	2.52 ± 0.62 b
Whiteness index (*WI*)	81.99 ± 0.34 a	83.87 ± 0.17 b	84.70 ± 0.19 c	84.63 ± 0.02 c	84.76 ± 0.22 c	84.71 ± 0.28 c	84.29 ± 0.09 c	84.94 ± 0.11 c	84.57 ± 0.12 c	84.47 ± 0.19 c
Complex modulus G* (Pa)	25,381.1 a	14,606.0 b	21,012.3 c	22,025.3 c	21,273.9 c	21,675.6 c	18,277.8 d	30,171.6 e	20,013.0 c	30,181.7 e
Loss tangent *tan δ* (-)	0.426 a	0.511 b	0.451 c	0.445 c	0.434 c	0.439 c	0.462 c	0.409 d	0.473 e	0.399 f
Hardness	10.78 ± 0.07 a	8.49 ± 0.24 b	10.02 ± 0.13 c	11.90 ± 0.15 d	11.33 ± 0.27 d	12.10 ± 0.28 e	14.19 ± 0.15 f	18.42 ± 0.02 h	11.67 ± 0.32 d	13.41 ± 0.07 g
Elasticity	10.82 ± 0.07 a	11.05 ± 0.15 b	10.95 ± 0.03 a	10.86 ± 0.01 a	10.82 ± 0.01 a	10.88 ± 0.13 a	10.81 ± 0.01 a	10.80 ± 0.04 a	10.85 ± 0.08 a	10.82 ± 0.03 a
Cohesiveness	0.58 ± 0.01 a	0.61 ± 0.01 b	0.53 ± 0.01 c	0.59 ± 0.02 a	0.49 ± 0.06 d	0.55 ± 0.02 c	0.61 ± 0.04 b	0.61 ± 0.04 b	0.59 ± 0.05 a	0.59 ± 0.05 a
Relative adhesiveness	0.10 ± 0.04 a	0.10 ± 0.02 a	0.10 ± 0.06 a	0.12 ± 0.48 a	0.10 ± 0.04 a	0.10 ± 0.04 a	0.08 ± 0.02 a	0.12 ± 0.08 a	0.08 ± 0.02 a	0.03 ± 0.01 a

* CS = Control sample; PC 1—9 samples of processed cheeses with different proportions of water content (1st dosage: 2nd dosage; PC 1 = 90:10; PC 2 = 80:20; PC 3 = 70:30; PC 4 = 60:40; PC 5 = 50:50; PC 6 = 40:60; PC 7 = 30:70; PC 8 = 20:80; PC 9 = 10:90; % *w*/*w*). Mean values with different letters in the row differ significantly (*p* < 0.05).

**Table 5:** Results of differential scanning calorimetry analysis for processed cheese manufactured using a two-stage water addition after being stored for 30 days at 6 ± 2 °C. The results are presented as mean ± SD.

Sample *	Cooling Cycle		Heating Cycle			
	T_p,cryst_ (°C)	∆H_cryst_ (J·g^−1^)	T_onset_ (°C)	T_p,m_ (°C)	∆H_fus_ (J·g^−1^)	W_f,s_ (%)
CS	−19.36 ± 1.24 a	71.64 ± 2.00 a	−9.05 ± 1.47 a	−3.26 ± 0.91 a	72.65 ± 1.57 a	21.78 ± 0.47 a
PC 1	−15.82 ± 2.62 b	105.66 ± 1.68 a	−7.19 ± 0.47 b	−2.55 ± 0.46 b	109.50 ± 0.89 b	32.83 ± 0.27 b
PC 2	−21.17 ± 2.09 c	54.328 ± 1.61 b	−10.71 ± 0.09 a	−4.60 ± 0.77 c	56.02 ± 0.22 a	16.80 ± 0.07 c
PC 3	−17.80 ± 1.32 d	72.45 ± 0.86 c	−8.56 ± 0.25 a	−2.94 ± 0.13 b	74.20 ± 0.63 a	22.25 ± 0.19 a
PC 4	−16.24 ± 0.37 e	98.81 ± 0.90 d	−6.80 ± 0.47 b	−2.23 ± 0.23 b	104.55 ± 1.63 b	31.35 ± 0.49 b
PC 5	−16.01 ± 2.83 e	100.40 ± 1.41 a	−6.87 ± 0.74 b	−2.40 ± 0.28 b	100.02 ± 1.70 b	29.99 ± 0.51 d
PC 6	−13.61 ± 0.66 f	117.65 ± 0.35 a	−6.32 ± 0.02 b	−1.95 ± 0.02 b	122.03 ± 1.15 b	36.59 ± 0.35 e
PC 7	−14.48 ± 1.48 h	104.028 ± 1.63 a	−7.06 ± 0.95 b	−2.28 ± 0.52 b	106.21 ± 1.10 b	31.85 ± 0.33 b
PC 8	−15.55 ± 0.69 b	75.918 ± 1.83 a	−7.70 ± 1.03 b	−3.14 ± 0.91 a	77.92 ± 1.36 a	23.36 ± 0.41 a
PC 9	−14.69 ± 0.18 i	107.95 ± 0.99 a	−6.68 ± 0.33 b	−1.93 ± 0.64 b	117.19 ± 0.64 b	35.14 ± 0.19 e

T_p,cryst_ = water crystallization peak temperature; ΔH_cryst_ = freezing enthalpy; T_onset_ = onset melting temperature; T_p,m_ = ice melting peak temperature; ΔH_fus_ = melting fusion enthalpy; W_f,s_ = freezable free and freezable bound water content. * CS = Control sample; PC 1—9 samples of processed cheeses with different proportions of water content (CS = control sample; PC 1—9 samples of processed cheeses with different proportions of water content (1st dosage: 2nd dosage; PC 1 = 90:10; PC 2 = 80:20; PC 3 = 70:30; PC 4 = 60:40; PC 5 = 50:50; PC 6 = 40:60; PC 7 = 30:70; PC 8 = 20:80; PC 9 = 10:90; % *w*/*w*)). Mean values with different letters in the column differ significantly (*p* < 0.05).

**Table 6:** Sensorial attributes of processed cheese samples for appearance, consistency, hardness, spreadability, flavor, and off-flavor evaluated by an expert panel (n = 12); values are expressed as the median ^1^.

Sample *	Appearance	Consistency	Hardness	Spreadability	Flavor	Off-Flavor
CS	1	4 a	4 a	4 a	1 a	1 a
PC 1	1	3 b	2 b	3 b	1 a	1 a
PC 2	1	3 b	3 c	3 b	1 a	1 a
PC 3	1	3 b	4 a	4 a	1 a	1 a
PC 4	1	3 b	4 a	4 a	1 a	1 a
PC 5	1	2 c	3 c	3 b	1 a	1 a
PC 6	1	4 a	4 a	4 a	1 a	1 a
PC 7	1	6 d	6 d	6 c	1 a	1 a
PC 8	1	4 d	4 a	4 a	1 a	1 a
PC 9	1	3 b	6 d	5 d	1 a	1 a

^1^ Appearance, consistency, hardness, spreadability and flavor: 1 = excellent; 3 = good; 5 = less good, 7 = unacceptable. Off-flavors: 1 = negligible, 4 = medium, 7 = excessive. Median values with different letters in the column differ significantly (*p* < 0.05). * CS = Control sample; PC 1—9 samples of processed cheeses with different proportions of water content (1st dosage–2nd dosage; PC 1 = 90:10; PC 2 = 80:20; PC 3 = 70:30; PC 4 = 60:40; PC 5 = 50:50; PC 6 = 40:60; PC 7 = 30:70; PC 8 = 20:80; PC 9 = 10:90; % *w*/*w*).

## Data Availability

The original contributions presented in this study are included in the article. Further inquiries can be directed to the corresponding author.
